# Quantifying long-term health and economic outcomes for survivors of group B Streptococcus invasive disease in infancy: protocol of a multi-country study in Argentina, India, Kenya, Mozambique and South Africa

**DOI:** 10.12688/gatesopenres.13185.2

**Published:** 2021-07-19

**Authors:** Proma Paul, Simon R. Procter, Ziyaad Dangor, Quique Bassat, Amina Abubakar, Sridhar Santhanam, Romina Libster, Bronner P. Gonçalves, Shabir A. Madhi, Azucena Bardají, Eva Mwangome, Adam Mabrouk, Hima B. John, Clara Sánchez Yanotti, Jaya Chandna, Pamela Sithole, Humberto Mucasse, Patrick V. Katana, Artemis Koukounari, Lois M. Harden, Celine Aerts, Azra Ghoor, Shannon Leahy, Sibongile Mbatha, Sarah Lowick, Sanjay G. Lala, Justina Bramugy, Charles Newton, A. K. M. Tanvir Hossain, Qazi Sadeq-ur Rahman, Philipp Lambach, Mark Jit, Joy E. Lawn

**Affiliations:** 1 Maternal, Adolescent, Reproductive & Child Health (MARCH) Centre, London School of Hygiene & Tropical Medicine, London, UK; 2 Department of Infectious Disease Epidemiology, London School of Hygiene & Tropical Medicine, London, UK; 3 Medical Research Council: Vaccines and Infectious Diseases Analytical Unit, Faculty of Health Sciences, University of the Witwatersrand, Johannesburg, South Africa; 4 ISGlobal, Hospital Clínic, Universitat de Barcelona, Barcelona, Spain; 5 Centro de Investigação em Saúde de Manhiça (CISM), Maputo, Mozambique; 6 ICREA, Barcelona, Spain; 7 Pediatric Infectious Diseases Unit, Pediatrics Department, Hospital Sant Joan de Déu (University of Barcelona), Barcelona, Spain; 8 Consorcio de Investigación Biomédica en Red de Epidemiología y Salud Pública (CIBERESP), Madrid, Spain; 9 Neuroscience Research Group, Department of Clinical Sciences, KEMRI-Wellcome Trust, Kilifi, Kenya; 10 Institute of Human Development, Aga Khan University, Nairobi, Kenya; 11 Neonatology Department, Christian Medical College, Vellore, India; 12 Fundación INFANT, Buenos Aires, Argentina; 13 National Technical and Scientific Research Council, Buenos Aires, Argentina; 14 Department of Science and Technology/National Research Foundation: Vaccine Preventable Diseases, Faculty of Health Sciences, University of the Witwatersrand, Johannesburg, South Africa; 15 Brain Function Research Group, School of Physiology, Faculty of Health Sciences, University of the Witwatersrand, Johannesburg, South Africa; 16 Department of Paediatrics and Child Health, Faculty of Health Sciences, University of the Witwatersrand, Johannesburg, South Africa; 17 Department of Psychiatry, Medical Sciences Division, University of Oxford, Oxford, UK; 18 Maternal and Child Health Division, International Centre for Diarrhoeal Disease Research, Bangladesh (icddr,b), Dhaka, Bangladesh; 19 Department of Immunization, Vaccines and Biologicals (IVB), World Health Organization, Geneva, Switzerland; 20 Modelling and Economics Unit, Public Health England, London, UK; 21 Division of Epidemiology and Biostatistics, School of Public Health, University of Hong Kong, Hong Kong SAR, China

**Keywords:** Group B streptococcus, meningitis, sepsis, infants, children, impairment, neurodevelopment, disability, economic, cost

## Abstract

Sepsis and meningitis due to invasive group B *Streptococcus* (iGBS) disease during early infancy is a leading cause of child mortality. Recent systematic estimates of the worldwide burden of GBS suggested that there are 319,000 cases of infant iGBS disease each year, and an estimated 147,000 stillbirths and young-infant deaths, with the highest burden occurring in Sub-Saharan Africa.  The following priority data gaps were highlighted: (1) long-term outcome data after infant iGBS, including mild disability, to calculate quality-adjusted life years (QALYs) or disability-adjusted life years (DALYs) and (2) economic burden for iGBS survivors and their families. Geographic data gaps were also noted with few studies from low- and middle- income countries (LMIC), where the GBS burden is estimated to be the highest. In this paper we present the protocol for a multi-country matched cohort study designed to estimate the risk of long-term neurodevelopmental impairment (NDI), socioemotional behaviors, and economic outcomes for children who survive invasive GBS disease in Argentina, India, Kenya, Mozambique, and South Africa. Children will be identified from health demographic surveillance systems, hospital records, and among participants of previous epidemiological studies. The children will be aged between 18 months to 17 years. A tablet-based custom-designed application will be used to capture data from direct assessment of the child and interviews with the main caregiver. In addition, a parallel sub-study will prospectively measure the acute costs of hospitalization due to neonatal sepsis or meningitis, irrespective of underlying etiology. In summary, these data are necessary to characterize the consequences of iGBS disease and enable the advancement of effective strategies for survivors to reach their developmental and economic potential. In particular, our study will inform the development of a full public health value proposition on maternal GBS immunization that is being coordinated by the World Health Organization.

## Introduction

The United Nations Sustainable Development Goals (SDGs) aim to complete the unfinished agenda for child survival and ensure that every child has the opportunity to thrive, including reaching their developmental potential ^1, 2^. While SDG3 continues to center on the reduction of neonatal and child mortality, SDG4 incorporates specific targets and indicators to address early childhood development (UN SDG, 2015). It is recognized that preventable infections, such as those that cause meningitis, neonatal sepsis, and pneumonia, are an important cause of neonatal and infant death ^3^. However, their contribution to neurodevelopmental impairment (NDI), which encompasses both developmental delay (two or more developmental domains in children ≤5 years old) and disability (impairment in a child’s physical, learning, language, or behavior function) has been under-appreciated. As child deaths are reduced in low- and middle-income countries (LMIC), neurodevelopmental impairment may increase, especially if access and quality of early childhood developmental programs is sub-optimal ^4, 5^.

Invasive group B *Streptococcus* (iGBS) disease during the first months of life is one of the infections that might have important long-term consequences for children. This infection often presents as sepsis or meningitis and was responsible for an estimated 90,000 (uncertainty range [UR]: 36,000-169,000) infant deaths in 2015 ^6^. Survivors of iGBS disease in early life may develop long-term NDI. Of 18 studies identified in a recent review of the risk of NDI in children with history of iGBS disease ^7^, only three were from middle-income countries and none were from low-income countries where the majority of iGBS disease cases occur. In these studies, NDI was defined as problems of body function and structure, such as significant deviations or loss in intellectual and/or motor, vision, or hearing impairment. The review concentrated on infants with GBS meningitis, highlighting a key data gap related to long-term adverse outcomes in infants who develop GBS-associated sepsis. Only a small number of older studies (primarily from the 1970s) reported NDI outcomes in children older than 2 years, which would have missed impairment outcomes that do not manifest until later in childhood. Although, a recent study from Denmark and the Netherlands has added to this data gap for high-income countries ^8^, data from LMIC remains a key gap.

Intrapartum antibiotic prophylaxis (IAP) has reduced the incidence of early-onset iGBS disease in some high-income countries ^9, 10^; however, this approach is less effective in preventing late-onset invasive disease ^11^ and thought not to significantly affect other consequences of maternal GBS colonization, notably GBS-associated stillbirths and preterm births. Maternal vaccination against GBS is a promising alternative that could protect both mothers and infants against iGBS disease. To guide investment in maternal vaccines targeting GBS, it is necessary to estimate the health and economic burden caused by the disease globally ^12^. Studies on mortality and morbidity due to GBS among pregnant and postpartum women, stillbirths, and infants have been recently reviewed and meta-analyzed ^13^. These reviews uncovered two major data gaps that significantly hinder these analyses: the lack of long-term follow-up data amongst survivors, which are needed to calculate generic health-related utility measures such as quality-adjusted life years (QALYs) or disability-adjusted life years (DALYs) to allow comparison with other diseases, and the lack of primary data regarding long-term economic consequences to households of children with a history of iGBS disease.

Data on the costs associated with iGBS disease, which are needed to inform cost-effectiveness analysis and investment decisions on the development and deployment of new vaccines, are scarce. We are only aware of a single study undertaken in the UK that directly assessed the economic costs of iGBS disease beyond the acute episode ^14^. This showed that over the first two years of life, the health and social care costs of infants with a history of iGBS disease were almost twice that of children with no history of iGBS disease. Although there are more studies that report on the acute costs of infant sepsis/meningitis, these are generally from high-income countries and are not GBS etiology specific ^15^. To better understand the life-course consequences of infant iGBS, data on health and economic outcomes needs to be collected in studies that include older children and adolescents across multiple settings.

In this paper, we present the protocol for a multi-country epidemiological study, coordinated by the London School of Hygiene & Tropical Medicine. The main aim of the study is to estimate the risk of NDI and socioemotional behaviors in children who survived neonatal or infant iGBS. The study will also measure long-term health related quality of life (HRQoL) and economic costs that arise as a consequence of iGBS, as well as the acute costs of sepsis and meningitis in young infants. Beyond the direct estimation of these outcomes in our study population, this study is also part of a collaboration with the World Health Organization (WHO) to inform development of a full public health value (FPHV) proposition for maternal vaccines against GBS ^16^.

### Research objectives

Our objectives in designing this study were:

**Objective 1 - Long-term neurodevelopmental impairment:**

**(a)** *Primary objectives:*
  1. To estimate the risk of moderate/severe NDI in children with history of iGBS disease in early-infancy, and to compare this with the risk in children with no known history of iGBS
  2. To estimate the risk of mild and moderate/severe socioemotional behavior outcomes in children with history of iGBS, and compare with risk in children with no known history of iGBS

**(b)** *Additional objectives:*
  1. To estimate the risk of mild impairment
  2. To estimate the risk of multi-domain and domain-specific neurodevelopmental impairment
  3. To estimate the risk of adverse growth outcomes (e.g., stunting, wasting)
  4. To estimate the risk of epilepsy

**Objective 2 – Long-term mortality:** To assess mortality beyond initial hospital-discharge among children who had iGBS.

**Objective 3 – Long-term economic costs and health-related quality of life:**

**(a)** *Long-term economic consequences*: To measure the long-term economic costs to the healthcare system, households and society associated with infant iGBS.
**(b)** *Health-related quality of life*: To collect information needed to calculate the difference in QALYs between children with a history of iGBS, and those with no history of iGBS.

**Objective 4 – Short-term economic consequences:** To estimate the costs to the healthcare system and households during acute episodes of sepsis and meningitis (irrespective of etiology) in neonates and young infants.

## Protocol

### Study design

***Long-term outcomes after iGBS disease in infancy (Objectives 1, 2, 3).*** We will use a matched cohort study design to collect data on NDI, socioemotional behavioral, and economic outcomes for survivors of iGBS in early infancy. Children with a history of infant iGBS (henceforth iGBS survivors), will be identified via hospital records in study sites, Health and Demographic Surveillance Systems (HDSS), or among participants of previous epidemiological studies. Children with no history of iGBS (henceforth, the non-iGBS comparison group) will be identified and matched to iGBS survivors based on sex and birth month and year. In Mozambique, children will also be matched on neighborhood location.

***Acute costs of neonatal sepsis and meningitis study (Objective 4).*** In addition to the main study measuring long-term economic outcomes, we will undertake a separate study to quantify the acute costs to the healthcare system and household linked to neonatal sepsis and meningitis. This study will involve a different study population: prospectively identified neonates admitted for clinically suspected sepsis or meningitis irrespective of the underlying etiology. Data on the costs associated with the period of acute hospitalization will be collected following discharge.

### Study settings and teams

Since the major data gap on the long-term outcomes of iGBS survivors is in LMICs, this study was designed to collect data in these settings, including at least one country per GBS high-burden region (Africa, Asia, Latin America). For this collaborative work, we shared a call for data through multiple channels in 2018, including targeting previous collaborators, experts and known GBS researchers, scientific conferences and meetings, as well as sending direct requests from WHO headquarters to country offices and placing posts on social media platforms to reach the widest number of people. With these various approaches we aimed to ensure better geographical representation than currently seen in the literature. Among those who responded, potential study sites were identified based on the following criteria: (a) sites that had at least 10 post-discharge surviving iGBS cases that could be enrolled; (b) sites that had neurodevelopmental follow-up data or the ability to collect this type of data in children aged at least 3 years; (c) sites where the expected loss to follow-up was <20%. From those who expressed an interest in joining this project and fulfilled the above criteria, research teams from Argentina, India, Kenya, Mozambique and South Africa agreed participate in this work and lead investigations locally ( Table 1).

**Table 1.** Description of collaborative research partners and study population participating in long-term and acute cost studies.

Country of data collection	Collaborative Institute(s)	Facility type	Long-term outcomes study (Objectives 1,2,3)			Acute cost study (Objective 4)	
			Identification of GBS- exposed children	Identification of GBS- unexposed children	Age at enrolment	Identification of meningitis/sepsis	Age at enrolment
Argentina	Fundación Infant, Buenos Aires, Argentina	2 Public hospitals in Tucuman area	Neonates admitted with GBS sepsis or meningitis from 2003– 2016	Primary Care Centers that belong to the Maternity Network	3–16 years	N/A	N/A
India	Christian Medical College (CMC) Vellore, Tamil Nadu, India	Academic and referral hospital at CMC Vellore	Hospital- delivered neonates admitted from 2004–2018	Hospital birth registry	18 months – 15 years	Sepsis with positive blood culture. Meningitis with either positive CSF culture or suggestive CSF counts or protein	0–89 days old
Kenya	KEMRI- Wellcome Trust, Kilifi, Kenya	Kilifi County Hospital / KEMRI	Admitted with GBS from 2007–2018	Health Demographic Surveillance System	1–12 years	Sepsis with positive: blood culture Meningitis with positive CSF culture or suggestive CSF counts or protein	
Mozambique	Barcelona Institute for Global Health, Barcelona, Spain	Manhiça District Hospital	Isolated during routine morbidity and microbiological surveillance conducted 2001–2018	Health Demographic Surveillance System	3–17 years	Sepsis with positive blood culture or clinically presumed sepsis Meningitis with positive CSF culture or suggestive CSF counts or protein; or clinically presumed meningitis	
	Manhiça Health Research Centre, Manhiça, Mozambique						
South Africa	Wits Health Consortium, Johannesburg, South Africa	3 Academic hospital in Johannesburg	Surveillance of the pediatric wards and microbiology services at the three hospitals from 2012– 2015	Unexposed children enrolled during a similar time-period as exposed children	5–7 years	Sepsis with positive blood culture Meningitis with positive CSF culture, latex agglutination, PCR or suggestive CSF counts	

In Argentina, the local study will be performed by the research organization Fundación Infant, in Buenos Aires; in Southeast India, the research activities are led by the Christian Medical College in Vellore; in Kilifi, Kenya, the work is being undertaken by KEMRI-Wellcome Trust Research Programme; in Mozambique, children are being recruited at the Manhiça Health Research Centre, in collaboration with the Barcelona Institute for Global Health; and in South Africa, the project is being led by the South African Medical Research Council Vaccines and Infectious Diseases Analytical Research Unit (VIDA).

### Long-term health outcomes after GBS invasive disease in infancy (Objectives 1, 2 & 3)

***Study populations.*** In the Kenya and Mozambique sites, which are also HDSS sites, parents of potential study participants, based on the HDSS database, will be contacted through standard recruitment procedures. Hospital records, which are linked to the HDSS database, will be used to identify all children who have been admitted with iGBS based on the case definition. The HDSS database will also be used to select matched non-iGBS children from the community. 

In Argentina and India, hospital-based databases will be used to identify potential iGBS survivors and non-iGBS children using standard practices established by each site. Hospital-based databases will be used to identify children who have been admitted with iGBS based on the case definition. Hospital-based birth registries will be used to select matched non-iGBS children. 

In South Africa, the same cohort of iGBS survivors and non-iGBS children from three epidemiological studies that were conducted between 2012 and 2015 will be contacted for re-enrolment. These participants are expected to be 5–7 years old and originally consented to be followed until the age of 5. Using the study database, parents or primary caregivers of these children will be contacted by phone for interest and be given information about participating in the new study.

***Case definition and exclusion criteria.*** Children with a previous diagnosis of either GBS meningitis or GBS sepsis in the first 90 days of life (days 0 - 89) will be recruited in these local epidemiological studies. Enrolment of children with a history of GBS sepsis is important to increase the, currently limited, number of studies with data on long-term disability post-GBS sepsis ^7^. Table 2 below summarizes the case definition and the clinical and microbiological eligibility criteria used by each study site for identification of exposed and unexposed children in this study.

In Argentina, India, Kenya, and Mozambique iGBS children and GBS unexposed children born early than 32 weeks of gestation are excluded. In South Africa, gestational age is not an exclusion criterion ( Table 2). 

**Table 2.** Definitions for the exposed (invasive GBS disease) and unexposed (non-iGBS) groups, and exclusion criteria used for recruitment for the long-term outcomes study (adapted from 17).

Definition	Exposed (iGBS) group		Unexposed (non-iGBS) group	Exclusion criteria
	Sepsis	Meningitis		
Argentina	Clinical signs of pSBI and/or GBS-positive blood culture or PCR or latex agglutination	Clinical signs of pSBI and [(GBS-positive CSF culture or PCR or latex agglutination) or (GBS-positive blood culture or PCR or latex agglutination and CSF leucocyte count of >20×10 ^6^/l)]	No clinical signs of pSBI and no known genetic disease	Very preterm (<32 weeks)
India			No clinical signs of pSBI	
Kenya				
Mozambique				
South Africa			No clinical signs of pSBI and not hospitalized in the first 3 months of life	No additional exclusion criteria

pSBI, possible serious bacterial infection; CSF, cerebrospinal fluid; PCR, polymerase chain reaction. pSBI definition: Any one of the following: a history of difficulty feeding, history of convulsions, movement only when stimulated, respiratory rate of 60 breaths per min or more, severe chest in-drawing, temperature ≥ 37.5°C or ≤35.5°C.

***Sample size and power calculation.*** The number of iGBS survivors included in the matched cohort study of long-term outcomes was based on the maximum number of cases expected to be identified, accounting for 20% being unreachable, ineligible or who refuse participation. The expected number of iGBS survivors for each site is summarized in Table 3. Based on anticipated recruitment of 200 iGBS survivors and a 1:3 ratio of matched non-iGBS children, and assuming a prevalence of our primary outcome (moderate/severe NDI) of 26% in iGBS survivors and 10% in the non-iGBS comparison group (based on a study of meningococcal serogroup B survivors ^18^), a pooled analysis would be able to detect this difference using a two-sided test of binomial proportions with 99% power at a 5% significance level. Mild developmental impairment is likely to be more prevalent among iGBS survivors, including those with sepsis. Our power to detect a difference in the risk of overall NDI (including mild NDI) would be 78%, assuming detection of 32% and 20% NDI in iGBS survivors ^7^ and non-iGBS children ^19^, respectively.

**Table 3.** Expected number of children with history of iGBS, by site, for the long-term outcomes study.

Site/Country	Expected number children with history of iGBS
Argentina	40
India	30
Kenya	50
Mozambique	40
South Africa	40

***Study procedures and data collection.*** Trained fieldworkers will contact the parents/primary caregivers of these potential participants about the study by phone (Argentina, India, South Africa) or in-person (India if phone contact information is not available, Kenya, Mozambique) and those contacted will be asked to make a one-time visit to the health facility with their child. Reasons for non-participation, such as migration, refusal or death will be recorded.

Children enrolled in the study and their main caregiver will receive an in-person assessment visit. Written informed consent will be obtained in-person either at the time of the initial house visit or before the in-person assessment visit. Only if appropriate consent/assent is obtained, will the child be enrolled in the study. 

At the in-person assessment visits, the following information will be collected:

- Questionnaire to collect participant details including birth and medical history, education, household demographic and socioeconomic data, as well as economic outcomes (for details on economic outcomes see section *Economic outcomes and health related quality of life*)
- Age-specific neurodevelopmental assessment tools including several domains (motor, vision, hearing, cognitive, language, socioemotional), an epilepsy screening questionnaire, and anthropometric measures (see section on *Assessment of developmental outcomes)*
- EQ-5D-3L questionnaires to assess the health-related quality-of-life (HRQoL) of study participants and their main caregiver (see section *Economic outcomes and health related quality of life*).

**Figure 1.**
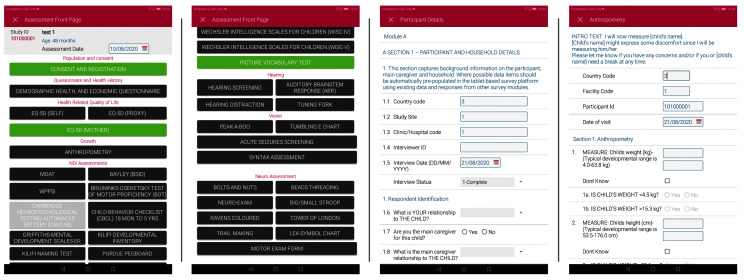
Screenshots from the customized data capture app.

Data will be collected on paper forms or using a customized app (developed in collaboration with icddr,b, Bangladesh). The customized Android tablet-based app includes questionnaires and neurodevelopment assessment tools, translated into local language where relevant ( Figure 1).

***Assessment of neurodevelopmental impairment (Objective 1).*** In this multi-country study, we will use several tools to diagnose NDI and socioemotional and behavioral outcomes in children over a wide age range from 18 months to 17 years old. The inclusion of children older than those enrolled in the majority of the previous epidemiological studies enables us to use more complex developmental assessments designed for older ages to detect specific mild NDI and other developmental delays. By including a wider age range, we can also better understand the developmental trajectory of infants who have survived iGBS. The developmental domains of interest are motor, vision, hearing, cognitive, language, and socioemotional; their definitions, by severity, are described in the *Data analysis section*. We will also explore growth outcomes and epilepsy.

The diagnostic tools used to identify NDI and other developmental measurements will be administered by experienced assessors, clinical psychologists and pediatricians, who will also perform clinical exams to identify impairment in hearing, motor and vision domains. Each local research team decided which neurodevelopmental assessment tools are appropriate for their setting, based on child’s age and cultural appropriateness or validation of the instrument and technical capacity of each site. There will be 26 different assessment tools and tests being used across the 6 neurodevelopmental domains, as well as anthropometric measurements for growth and an epilepsy screening questionnaire. Table 4 shows the matrix of assessments for each developmental domain, by age category and study site. When a need for further assessment and clinical management is identified, children will be appropriately referred into each site’s existing referral systems. 

**Table 4.** Neurodevelopment assessment tools and other developmental assessment measures, by site and age.

	Sites				
	Argentina	India	Kenya	Mozambique	South Africa
Motor					
< 5 years old	Pediatric clinical exam	BSID * BOT ^‡^	KDI *	MDAT	N/A
5 – < 10 years old		BOT ^‡^	Bolts and Nuts Bead Threading	CANTAB	GMDS- ER
≥ 10 years old			Stork Balance Ball Balance		N/A
Cognition					
< 5 years old	WPPSI ^†^	BSID * WPPSI ^†^	KDI * Big/small stroop	MDAT	N/A
5 – < 10 years old	WPPSI ^†^ WISC 4 ^¥^	WPPSI ^†^ WISC 5 ^¥^	RCPM Tower of London	CANTAB	GMDS- ER
≥ 10 years old	WISC 4 ^¥^	WISC 5 ^¥^	RCPM Trail Making		N/A
Language					
< 5 years old	WPPSI ^†^	BSID *	KDI * PVT, Measures of pragmatics	MDAT	N/A
5 – < 10 years old	WPPSI ^†^	WPPSI ^†^	Kilifi Naming Test Measure of Pragmatics and Syntax	CANTAB	GMDS- ER
≥ 10 years old	WISC 4 ^¥^	WISC 5 ^¥^			N/A
Hearing					
≤ 4 years old	Screening test: Distraction test Further testing: ABR				N/A
> 4 years old	Screening test: Tuning fork, Diagnostic memory audiometer Further testing: ABR				
Vision					
≤ 3 years old	LEA symbols Chart or Picture chart				N/A
> 3 years old	Visual acuity app/Tumbling E chart/Snellen chart				
Socioemotional					
≤ 6 years old	CBCL-preschool				
> 6 years old	CBCL- school aged				
Epilepsy					
All ages	Epilepsy Screening Questionnaire (ESQ)				

*BSID assessment up to 42 months. ^†^ WPPSI assessment in Argentina 3-7 years. WPPSI assessment in India 4-7 years. ^‡^ BOT assessment ≥4 years. ^¥^ WISC 4 and WISC 5 assessment ≥7 years. ABR, auditory brainstem response; BOT, Bruininks-Oseretsky Test; BSID, Bayley Scales of Infant and Toddler Development; CANTAB, Cambridge Neuropsychological Test Automated Battery; CBCL, Child Behavior Checklist; GMDS-ER, Griffiths Mental Development Scales – Extended Revised; KDI, Kilifi Developmental Inventory; MDAT, Malawi Developmental Assessment Tool; PVT, Picture Vocabulary test; RCPM, Raven’s colored progressive matrices; WISC, Wechsler Abbreviated Scale of Intelligence; WPSSI, Wechsler Preschool and Primary Scales of Intelligence.

***Mortality outcome (Objective 2).*** Whenever feasible, data on the cause of death will be captured through a variety of methods, including by reviewing medical records, verbal autopsy reports and interviews with parents, for iGBS survivors who died after the acute episode and before enrolment and for matched non-iGBS group who died. In Kenya, the list of iGBS survivors who could potentially be enrolled in the study only included children alive at the time of enrolment, therefore data on early mortality post-iGBS disease will not be collected in these sites.

***Long-term economic costs and health-related quality of life outcomes (Objective 3).*** There are only limited data available on the economic consequences of iGBS, which cover only healthcare costs in the first two years of life ^15^. In this study, we will collect information on variables that will allow comparisons of economic outcomes in families of iGBS survivors versus families of the non-iGBS comparison group. These data will also be used to inform future economic analyses that will be performed to assess the value of maternal vaccines against iGBS.

A summary of key economic variables is shown in Table 5. Information collected will include details of the monthly household income and expenditure, participating children’s healthcare utilization, out-of-pocket payments, and any expenditure on social care or special education in the 12 months preceding study enrolment. Additionally, information will be collected on time spent by the main caregiver providing informal care to the participant, as well as information on the costs of coping strategies, such as borrowing and asset sales. Information on the HRQoL of both the participant and the main caregiver will be collected using an EQ-5D-3L questionnaire in three countries where country-approved translations are available: Argentina (Spanish), India (English, Telugu, Tamil) and South Africa (English, Zulu).

**Table 5.** Economic data to be collected as part of the long-term and acute cost studies.

Category	Measures	Acute	Long- term
**Participant characteristics**	Participant details including date of birth, gender and ethnic group. Relevant medical history including HIV status, gestational age at birth, and birthweight. Educational status.	Yes	Yes
**Caregiver characteristics**	Age, gender, and relationship to the child. Education level and occupation.	Yes	Yes
**Household characteristics**	Number, relationship to patient and ages of other household occupants. Education level and occupation of head of household and mother. Household income and welfare received. House-hold socioeconomic status based on local asset index. Household location (urban / rural).	Yes	Yes
**Healthcare resource use** **during acute episode**	Length-of-stay by bed type (e.g. ICU vs general bed) and days of supportive care (e.g. ventilation, NG tube, Oxygen, IV fluids). Diagnostics (e.g. lumbar puncture, blood tests, blood/CSF cultures, diagnostic imaging) and medicine use.	Yes	No
**Household expenditure**	Total household expenditure including separate expenditure on health, transport, education, and food.	Yes	Yes
**Participant HRQoL**	For long-term cohorts a self-reported EQ-5D-3L for children aged 11 and over; a proxy-reported EQ-5D-3L for children aged 3 to 11. For the acute cost study, a proxy-reported Visual Analogue Scale.	Yes	Yes
**Caregiver HRQoL**	A self-reported EQ-5D-3L.	Yes	Yes
**Participant healthcare &** **out-of-pocket payments**	Number of visits and number of days admitted to a hospital. Number of visits to healthcare facilities or traditional healers, and home visits by community healthcare professionals. Out-of-pocket payments on healthcare including drug costs, travel and accommodation and caregiver time spent accompanying participants to hospital.	Yes	Yes
**Participant social care & out-** **of-pocket payments**	Use of and cost of special educational services. Use of and cost of professional care in the home. Provision and cost of any home modifications.	No	Yes
**Informal caregiving**	Time spent by the main caregiver providing care to the participant. Amount of paid work, subsistence work, housework foregone due to caregiving.	No	Yes
**Cost of coping**	Borrowing to cover healthcare and social care costs, or as the result of being unable to work. Value of assets sold to cover costs. Other coping mechanisms.	Yes	Yes

### Acute costs of neonatal sepsis and meningitis (Objective 4)

***Case definition for neonatal sepsis and meningitis.*** To be able to collect data on acute costs, both cases of severe neonatal infection linked to GBS and cases of severe neonatal infection due to other bacteria will be enrolled, as the number of confirmed iGBS cases per hospital is anticipated to be small over the duration of our study. Participants will be babies admitted with a diagnosis of clinically suspected neonatal infection (sepsis or meningitis) combined with isolation of a pathogenic microbiological agent by culture or detection by polymerase chain reaction (PCR) in a normally sterile site (blood/CSF) on day 0 – 89 of an infant’s life. Babies born at <32 weeks of gestational age, born with severe congenital abnormalities, or with culture positive results only for organisms considered to be contaminants or skin commensals will be excluded ( Table 6). In Mozambique the cases will be defined based on clinically suspected sepsis or meningitis because the number of bacteriologically confirmed cases is anticipated to be low due to the size of the hospital.

**Table 6.** Selected organisms considered possible contaminants or skin commensals for neonatal infection (non-exhaustive list).

Excluded organism
Coagulase-negative *Staphylococcus*
*Bacillus* spp.
*Micrococcus* spp.
*Corynebacterium* spp.
*Propionibacterium* spp.
Diphtheroids
*Aerococci*
*Brevundimonas vesicularis*
*Ochrobactrum anthropi*
*Staphylococcus saprophyticus*
*Burckholderia*/ NFGNB (if in first three days of life)
*Enterococcus* (if baby asymptomatic)
Cultures which show poly-microbial growth (unless baby has had abdominal surgery / or if it includes GBS/E coli)
Any bacterium which shows growth after 72 hours of life

***Sample size.*** At least 20 participants will be recruited in each of the four sites. The sample size of 20 per site was set as a practical minimum, considering both available resources and also a consensus by local research teams that this would be a feasible number to capture given the anticipated number of neonatal infections within the timeframe of data collection activities. 

***Study procedures.*** Participants will be identified prospectively either on admission or using clinical databases. Additionally, in India recent cases (within three months before the start of the study) will also be identified retrospectively from clinical records. Details on hospital resource use will be collected from medical records, including information on length-of-stay, type of hospital bed, and details of any drugs, diagnostic tests and surgical procedures. To capture the wider impacts of a participant's hospitalization, a questionnaire will be administered to the main caregiver, either at time of discharge, or by follow-up as soon as possible after discharge, to collect details on household demographics and economic impact. This will include any out-of-pocket payments, costs related to travel, accommodation, and caregiver time. Data will be gathered on the main caregiver’s HRQoL using an EQ-5D-3L questionnaire and the main caregiver will be asked to estimate their child’s HRQoL during their time in hospital using a Visual Analog Scale (VAS). The main caregiver will be encouraged to accompany the child to the assessment visit, but in the cases where they do not then these sections of the questionnaire will not be completed. No questionnaires will be administered in the case that a participating child dies in hospital, but data will still be collected from hospital records.

### Data management

Data will be stored on secure servers locally after the end of the study, and anonymized data will be transferred to the team at the London School of Hygiene & Tropical Medicine, where data from different countries will be pooled. Analyses will be conducted jointly by all study partners.

### Analysis plan

***Objective 1 - Long-term neurodevelopmental impairment.*** To allow comparison between the different neurodevelopmental assessments being used in each of the five sites, we will undertake a mapping activity across all 26 tools by age bands (1–4, 5–9, 10+). The age bands are constructed based on key periods of development ^17^. We will map similar constructs across the different assessments; e.g., gross motor measurements from all relevant tools will be mapped against each other allowing us to compare gross motor development across sites. We will do this for the following domains; gross motor, fine motor, cognitive and language. We will also do a similar mapping activity between the preschool and school-aged CBCL for the socioemotional and behavioral outcomes (e.g., anxiety, ADHD, and autism). Definitions domain-specific neurodevelopmental impairment and severity are described in Table 7.

In India, Kenya, Mozambique, and South Africa, motor and cognitive scores will be normalized using standard reference populations by assessment and site. In Argentina, where motor impairment is being assessed through a clinical exam, description of functional impact will be used.

**Table 7.** Definitions of growth and domain specific neurodevelopmental impairment severity.

Domain and severity		Severity definition used in this study
**Motor**	Mild	Motor for age Z-score -1 to -2 SD for test OR outside normal range of standardized motor score for mild classification OR no functional motor impairment from physical exam
	Moderate	Motor for age Z-score -2 to -3 SD for test OR outside normal range of standardized motor score for moderate classification OR moderate functional motor impairment from physical exam
	Severe	Motor for age Z-score ≤3 SD for test OR outside normal range of standardized motor score for severe classification OR moderate functional motor impairment from physical exam such as cerebral palsy
**Intellectual**	Mild	Cognitive for age Z-score -1 to -2 SD for test (DQ 70-84)
	Moderate	Cognitive for age Z-score -2 to -3 SD for test (DQ 55-69)
	Severe	Cognitive for age Z- score ≤3 for test (DQ <55)
**Language**	Mild	Language for age Z-score -1 to -2 SD for test OR outside normal range of standardized language score for mild classification
	Moderate	Language for age Z-score -2 to -3 SD for test OR outside normal range of standardized language score for moderate classification
	Severe	Language for age Z-score ≤3 SD for test OR outside normal range of standardized language score for severe classification
**Vision**	Mild	Visual acuity in best eye <6/12 but better or corresponding visual field loss
	Moderate	Visual acuity in best eye between 6/18 and 6/60, or corresponding visual field loss
	Severe	Visual acuity in best eye between 6/60 and 3/60, or corresponding visual field loss
	Blindness	Visual acuity in best eye <3/60, or corresponding visual field loss
**Hearing**	Mild	Audiometric hearing threshold level 26–30 decibel
	Moderate	Audiometric hearing threshold level 31–64.9 decibel
	Severe or deafness	Audiometric hearing threshold level ≥65 decibel
**Socioemotional/** **behavioral**	Mild	CBCL scores within borderline clinical range in at least one domain of the problem scales
	Moderate or severe	CBCL scores within clinical range ≥1 domain(s) of the problem scales
**Epilepsy**		Had at least one seizure in the last month
**Growth**	Stunted	Height for age Z-score < -2
	Underweight	Weight for age Z-score < -2
	Head circumference	Head circumference for age Z-score < -2

Vision impairment will be defined using WHO categories of mild (visual acuity in best eye ≤6/12), moderate (visual acuity in best eye ≤6/18 and >6/60), severe (visual acuity in best eye ≤6/60 and >3/60), and blindness (visual acuity in best eye ≤3/60) ^20, 21^.

Any hearing impairment will be defined as an unaided hearing threshold in the best ear of >26 decibels and further categorized into mild (audiometric hearing threshold level 26–30 decibels), moderate (threshold level 31–60 decibel), and severe/deafness (threshold level >60 decibel) ^20, 22^. In South Africa and Mozambique, screening tests will be used first to identify any individual with any potential hearing impairment. Results from further diagnostic tests will be used to classify into impairment severity as categorized above.

Socioemotional behavior measures will be defined in all sites using the CBCL assessment. The main scoring is based on a principal components analysis that grouped sets of behaviors into different syndrome scales: (1) internalizing problem scales, which include anxious/depressed, withdrawn-depressed, and somatic complaints scores; and (2) externalizing problem scales, which includes rule-breaking and aggressive behavior. There is also a total problem score which is the sum of all the items. Each syndrome, internalizing and externalizing problem score, and total score can be categorized into normal (<93rd percentile), borderline (93rd-97th percentile), or clinical behavior (>97th percentile) based on the same normative samples to create standard scores based on sex and age for all sites.

### Primary outcomes

There are 2 primary outcomes in this study, moderate/severe NDI and moderate/severe behavioral outcomes.

Moderate/severe NDI will be defined as:

- Score of >2 SD below the standardized reference mean in cognition AND/OR motor composite measures
- AND/OR hearing loss
- AND/OR vision loss

Moderate/severe behavioral outcomes will be defined by scores within clinical ranges of ≥1 domain(s) of the problem scales.

### Additional outcomes

Mild NDI (including socioemotional behavior outcomes) will be defined as:

- Score of 1-2 SD below the standardized reference mean in cognition AND/OR motor composite measures
- AND/OR mild hearing loss
- AND/OR mild vision loss
- AND/OR borderline clinical range in at least one domain of the problem scales from the CBCL

We will further assign individuals into the following multi-domain impairment categories based on severity (adapted from 23):

- Mild if child is classified as mildly impaired in ≤2 domains
- Moderate if child is classified as mildly impaired in 3 domains OR classified as moderately impaired in 1 domain & classified as mildly impaired in 2 domains
- Severe if child is classified as moderately impaired in ≥2 moderate domains OR severely impaired in ≥2 domains

The distribution of mild and moderate/severe neurodevelopmental outcomes will be summarized for children with history of iGBS disease and the non-iGBS comparison group and further stratified by clinical syndrome (sepsis and meningitis). We will test the association between history of iGBS disease in early-infancy and moderate/severe NDI in a pooled analysis using a logistic regression accounting for matching factors of age and sex. As gestational age is likely to be an important confounder, we will adjust for this. We will also adjust for other known confounders (e.g., SES, maternal education), if the data allows.

***Objective 2 - Mortality.*** For iGBS survivors, and their matched non-iGBS comparison group, that were reachable (i.e. for whom we have information), we will describe the proportion of children who died before enrolment in each site. Where available, we will also describe the causes of death in both iGBS and non-iGBS groups.

***Objective 3 - Long-term economic and health-related quality of life.*** We will assess the impact of iGBS on economic outcomes including healthcare utilization and costs, household out-of-pocket payments, household income and social care payments, and time spent by the main caregiver on informal care. The cost of hospital stays and attending outpatient clinics will be estimated using published unit costs (e.g. WHO-CHOICE) ^24^. We will compare these outcomes between the iGBS and non-iGBS groups in each study site. If data allow, we will also analyze differences in costs and healthcare utilization linked to NDI. Information from EQ-5D-3L questionnaires will be used to estimate differences in QALYs of both children and their caregivers associated with a history of iGBS disease.

***Objective 4 – Acute costs of neonatal sepsis and meningitis.*** Data from the sub-study on acute costs will be used to estimate the average length-of-stay, use of supportive care, drugs and diagnostics, during hospitalization for the acute neonatal sepsis/meningitis episode. These data will also be used to calculate the overall cost per episode. Other variables will be presented descriptively to characterize the impact of severe neonatal infection.

### Ethics

Written informed consent will be obtained from parents or guardians. Whenever appropriate, based on local guidelines, assent will also be obtained from children participating in the study. The overarching protocol for this multi-country observational study was granted ethical approval at the London School of Hygiene & Tropical Medicine (approval number 16246). Institutional review boards in each of the operating countries granted ethics approval (Argentina approval number Protocol EGB-1, India approval numbers 11723 (CMC Vellore), 2019–7034 (ICMR); Kenya approval number SERU/CGMR-C/164/3882; Mozambique approval numbers 98/CNBS/2019; South Africa approval number M190241), as well as the institutional review board of the World Health Organization (approval number ERC.0003169).

## Discussion

This multi-country study will provide new data on the consequences of iGBS, which is responsible for significant morbidity, disability and mortality in infants ^10, 19^. In particular, we will provide novel data on NDI and socioemotional and behavior outcomes, especially in LMIC contexts. Previous reviews have not included any low-income country data, outcomes due to GBS-associated sepsis, or mild NDI ^17^. Mild NDI may be common, impact families and societies, and are required to estimate DALYs, which are widely used as metrics to set priorities for resource allocation.

An important strength of this study is the inclusion of older children (3–17 years) representing three continents (Latin America, Africa, Asia), which currently have limited local data on NDI, socioemotional behavior outcomes, and wider socioeconomic consequences following iGBS disease. This will allow better understanding of the geographic variability on the risk of long-term disability linked to GBS. No previous studies have reported on the potential long-term (>2 years) consequences of GBS in these countries ^7^. Furthermore, our study population will include both children who developed sepsis and meningitis. Some studies suggest that severe NDI might be lower in children developing sepsis compared to meningitis ^25, 26^. However, since sepsis is more common among neonates with serious bacterial infection in LMIC settings ^5^, even mild NDI could make an important contribution to the overall morbidity of iGBS. In addition to the risk of long-term morbidity, iGBS may also lead to excess mortality after the acute episode. Although our study is not powered to compare mortality risk in iGBS survivors versus those without history of iGBS, we will be able to describe mortality and causes of deaths in four of five sites.

Another strength of our study is the collection of primary data on healthcare use, income, and HRQoL across multiple countries, which will enable us to identify where iGBS disease may lead to worse outcomes. Adverse economic outcomes due to iGBS are thought to be likely, for example costs linked to sequelae that necessitates frequent healthcare utilization, costly household adaptations, and additional time spent on caregiving to support a child with disabilities ^23, 27^. However, to our knowledge, only one study in the UK has directly measured the economic costs of iGBS. In that study, where children were followed-up to the age of two, the average health and social care costs were substantially higher amongst those with history of iGBS ^14^.

A major challenge of this study is the use of different developmental assessment tools in each country, and the complexity of combining multi-domain and neurodevelopmental outcomes for different age bands and multiple tests. We will try to ensure measurement equivalence and comparability of the NDI outcomes between different ages, assessment tools and sites through domain mapping of assessment tools, before individual-level data from each site is further combined for analysis.

A further challenge is the impact that the global coronavirus (COVID-19) pandemic will have on recruitment and research activities, which are not clear. We will continue to assess the situation and are working closely with research teams from each and collaborative institutes to safely undertake field activities in line with each country’s guidelines.

As well as the direct analysis of the data described in this protocol, findings from this study will also be used to update previous morbidity estimates of the global burden of infant iGBS disease ^13^, adding relevant data on long-term outcomes. This will include information to estimate lifetime disability, including risk of NDI, and societal impacts following GBS-related sepsis and meningitis. These additional morbidity estimates, combined with the previously published data on mortality and morbidity of pregnant and postnatal women and stillbirths, along with other literature, will serve as data inputs for both mother and infant GBS disease to update estimates of the overall public health burden of iGBS. Disease burden estimates will be translated into DALYs incurred based on the latest available epidemiologic data, while responses to the quality of life instruments combined with mortality data will be used to estimate QALYs lost. These estimates will be an important input feeding into future cost-effectiveness analyses.

These findings will contribute to the WHO-led development of a full public health value proposition for GBS maternal immunization to inform strategic planning of GBS vaccine research, development, and future implementation. Decision making by multiple stakeholders in the GBS vaccine development process, including research funders, manufacturers, donors and national governments, will be shaped by these findings. Data generated from this study will be linked with research outputs providing more regional and country specific details, allowing countries to utilize the findings in their own context. This will help reduce the translational, marketing, and implementation gaps for the development and introduction of a new GBS vaccine in LMICs which experience some of the highest disease burden.

Most of the mortality and morbidity of iGBS occurs in low-resource settings where there continues to be a paucity of data. As well as the limited epidemiological and clinical data, there are major gaps in data on the economic burden of both short-term and the long-term effects of iGBS. Our study will address limitations in the data currently available, providing new data on epidemiological and economic outcomes are needed to get a more complete picture on the consequences of iGBS for individuals and their families. Coordinated data collection across different settings together with harmonized analysis approaches, will maximize the value of the collected data. The results of our study will support development and investment in cost-effective strategies to minimize the iGBS burden and improve the chances for children to survive, thrive and reach their developmental and economic potential.

## Data availability

No data are associated with this article.

## Author information

Proma Paul and Simon R. Procter are joint-first authors; Mark Jit and Joy E. Lawn are joint-senior authors.
