# Supplementary material for: Quantifying long-term health and economic outcomes for survivors of group B Streptococcus invasive disease in infancy: protocol of a multi-country study in Argentina, India, Kenya, Mozambique and South Africa
Source: Gates Open Res. 2021 Jul 19;4:138. Originally published 2020 Sep 23. [Version 2] doi: 10.12688/gatesopenres.13185.2 (PMC8313848; doi:10.12688/gatesopenres.13185.2)
Supplement: Peer review history

# Peer review: Reviewer response for version 2

**DOI:** 10.21956/gatesopenres.14539.r30894

**Published:** 2021-07-26

**Authors:** David Kaslow

**Affiliations:** 1 Essential Medicines and Center for Vaccine Innovation and Access, PATH, Seattle, WA, USA

With thanks to the authors for their responses; no further comments.

Is the study design appropriate for the research question?

Partly

Is the rationale for, and objectives of, the study clearly described?

Yes

Are sufficient details of the methods provided to allow replication by others?

Yes

Are the datasets clearly presented in a useable and accessible format?

Yes

Reviewer Expertise:

vaccine and drug development and introduction

I confirm that I have read this submission and believe that I have an appropriate level of expertise to confirm that it is of an acceptable scientific standard.

# Peer review: Reviewer response for version 1

**DOI:** 10.21956/gatesopenres.14390.r30611

**Published:** 2021-05-28

**Authors:** Robert Simon Heyderman

**Affiliations:** 1 NIHR Global Health Mucosal Pathogens Research Unit @UCL, University College London, London, UK

This protocol describes an observational cohort study that will address two critical, largely unexplored, questions across a range of lower and middle-income countries: the long-term outcome of infant iGBS; and the economic burden for iGBS survivors and their families. The protocol is likely to deliver invaluable information that will impact on policy and practice with strong alignment to the WHO Defeating Meningitis agenda. I have several comments that could further improve the protocol and the outputs:

1. **Participant recruitment bias**: there will be survivor bias (even post-hospital discharge) and the potential for children with significant morbidities to be unavailable because they have been moved away from the study sites. The investigators should consider how they will account for this in their recruitment strategy and analysis.
2. **School attendance and specialist intervention bias**: given the breadth of settings, the investigators should state how they will ascertain and account for differences in schools and specialist services available to the participants at different sites.
3. **Matching of case controls**: will the controls come from the same geographic area/ socioeconomic background as the cases?
4. **Sample size:** outside the DSS populations, it seems optimistic that only 20% will be unreachable. Do the authors have any data to support this assertion?
5. **Definition of long-term mortality**: given that there will be different hospital discharge practices at the different sites, the current definition of long-term mortality is subject to this bias. Would it be better to set an age limit e.g. mortality beyond 3 months of age?
6. **HIV status**: particularly in South Africa and Mozambique, this ideally should be tested as part of this study in the cases and controls. The plan for status ascertainment should be made clear in the protocol.
7. **Limitations section**: there should be a summary of the limitations of this study design.
8. **Data confidentiality**: there should be a summary of how this will be addressed.
9. **Statistical analysis plan** (SAP): ideally a draft SAP should be included as an appendix but at the very least, there should be a commitment in the protocol to lodge an a priori SAP before analysis is undertaken. This should include an indication of what stratified and sensitivity analyses will be undertaken.
10. **Community Engagement & Involvement**: the authors should state how the local communities will be involved in this research.
11. **Results dissemination:** the authors should state how the results from this protocol will be disseminated.

**Minor comments**:

- a) Lab confirmation of sepsis: I am not aware that latex agglutination has been sufficiently validated on blood. Can the authors provide supporting evidence.
- b) Sepsis vs. meningitis: will there be a requirement for all sepsis cases to have had a diagnostic LP? If not, this should be clarified in the text.
- c) Child assent: at what age will assent for participation in the study be sort?

Is the study design appropriate for the research question?

Yes

Is the rationale for, and objectives of, the study clearly described?

Yes

Are sufficient details of the methods provided to allow replication by others?

Partly

Are the datasets clearly presented in a useable and accessible format?

Yes

Reviewer Expertise:

My interests span the basic biology, epidemiology, genomics and prevention of a range of mucosal pathogens including GBS.

I confirm that I have read this submission and believe that I have an appropriate level of expertise to confirm that it is of an acceptable scientific standard, however I have significant reservations, as outlined above.

# Peer review: Reviewer response for version 1

**DOI:** 10.21956/gatesopenres.14390.r30610

**Published:** 2021-04-26

**Authors:** Tina Perme

**Affiliations:** 1 Department of Perinatology, University Medical Centre Ljubljana, Ljubljana, Slovenia

The researchers describe this multi-centre protocol to assess long-term health and economic impact of invasive GBS disease clearly and concisely. The aim of this study to provide data with which to influence the development of effective strategies for coping with disease sequelae and for the development of effective immunization is convincing.

 However, there are some ambiguities regarding inclusion and exclusion criteria which need addressing if they have not yet been sufficiently provided in the full study protocol. The researchers state that the comparison group will be matched to GBS survivors based on sex and birth month and year. However, especially in low-income settings, there are many other potentially threatening infections or diseases that can influence socio-emotional, behavioral and economic outcomes for survivors. That needs to be considered and control group potentially adjusted to account for this other possible confounding factor.

 Additionally, as neurodevelopmental impairment is so differently assessed throughout the different centres, care must be taken to ensure that the outcomes provided are comparable between different study sites.

 In summary, this multi-centre study has the potential for providing important data regarding the burden of invasive GBS disease in low-income countries and influencing future strategies for coping with this infection.

Is the study design appropriate for the research question?

Partly

Is the rationale for, and objectives of, the study clearly described?

Yes

Are sufficient details of the methods provided to allow replication by others?

Yes

Are the datasets clearly presented in a useable and accessible format?

Yes

Reviewer Expertise:

Neonatology, microbiology, infectious diseases

I confirm that I have read this submission and believe that I have an appropriate level of expertise to confirm that it is of an acceptable scientific standard, however I have significant reservations, as outlined above.

# Response

**Published:** 2021-05-26

**Authors:** Simon Procter

We thank the reviewer for their comments. Our response to the specific points raised are below.

**(1) However, there are some ambiguities regarding inclusion and exclusion criteria which need addressing if they have not yet been sufficiently provided in the full study protocol. The researchers state that the comparison group will be matched to GBS survivors based on sex and birth month and year. However, especially in low-income settings, there are many other potentially threatening infections or diseases that can influence socio-emotional, behavioral and economic outcomes for survivors. That needs to be considered and control group potentially adjusted to account for this other possible confounding factor.**

 Response: Thank you. For all study participants we collect data on prior medical history but this does not include the history of other neonatal infections. We agree that clarity on the inclusion/exclusion criteria is fundamental, we have clarified this by revising table 2 to include definitions for unexposed (non-GBS) children and additional exclusion criteria related to gestational age. We also added the following sentence to the manuscript after the case definition:

 “ **In Argentina, India, Kenya, and Mozambique iGBS children and GBS unexposed children born earlier than 32 weeks of gestation are excluded.  In South Africa, gestational age is not an exclusion criterion.**”

 NDI that may result from other infections and disease will form part of the background risk of NDI in our study settings. While we are not collecting data on these other aetiological causes of NDI, if numbers allow we will examine the influence of other potential confounders such as prematurity, SES and maternal education using multivariable regression.

**(2) Additionally, as neurodevelopmental impairment is so differently assessed throughout the different centres, care must be taken to ensure that the outcomes provided are comparable between different study sites.**

 Response: This is indeed a challenge in our study, and for most studies that aim to assess and compare diverse neurodevelopmental outcomes across settings. We highlight this in our discussion, where we say: “A major challenge of this study is the use of different developmental assessment tools in each country, and the complexity of combining multi-domain and neurodevelopmental outcomes for different age bands and multiple tests.” To ensure comparability as far as possible we will work with clinical experts in the individual country teams to map the different neurodevelopmental outcomes across the different tools for greater comparability. As described in the protocol, domain definitions (e.g., cognition, motor, language) will be defined following the definitions used in the Global Burden of Disease (GBD). Then clinical experts will review each of the tools to determine how the full tool or subscales within the tool best aligns with the GBD definitions. For our our analysis where we will combine data on the severity and across domains of impairments we are following a similar approach to the GBD ( *Blencowe H, Vos T, Lee AC, Philips R, Lozano R, Alvarado MR, Cousens S, Lawn JE. Estimates of neonatal morbidities and disabilities at regional and global levels for 2010: introduction, methods overview, and relevant findings from the Global Burden of Disease study. Pediatr Res. 2013 Dec;74 Suppl 1(Suppl 1):4-16. doi: 10.1038/pr.2013.203).*

# Peer review: Reviewer response for version 1

**DOI:** 10.21956/gatesopenres.14390.r29649

**Published:** 2020-10-13

**Authors:** David Kaslow

**Affiliations:** 1 Essential Medicines and Center for Vaccine Innovation and Access, PATH, Seattle, WA, USA

The description of a multi-country protocol to quantify the acute costs and the long-term health and economic outcomes of survivors of invasive GBS disease is clear, concise, and timely. The rationale, that the results of this study provided essential input to a full value of vaccine assessment (aka full public health value preposition, FPHVP), is also clear and convincing.

 Two major questions come to this reviewer's mind when reading this manuscript:

1. Sample size and power calculation: this description appears to describe an approach for determining whether there is a statistically significant difference between the iGBS survivors group and the the matched non-iGBS control children for certain specific outcomes. That there is a statistically significant difference between the two groups is certainly of some interest, but would seem not to be the critical input data for a FPHVP assessment. Rather it would seem that the critical information would be: 1) an absolute point estimate and confidence intervals thereof of burden of disease outcomes that are the major value drivers in the FPHVP; and 2) the excess burden of disease (see point 2 below) in iGBS survivors that are the major value drivers in the FPHVP. Perhaps the data analyses and statistical methodology that support these two estimates, with confidence intervals, is in the protocol, but if not, the authors should be encouraged to add and describe those items.
2. Inclusion and exclusion criteria: Again, perhaps the information is provided in the protocol; however, it is unclear what the inclusion and exclusion criteria of selecting iGBS survivors, and equally important the case controls, with respect to other potential causes of the endpoints being measured. For example, NDI is multifactorial, including both genetic and infectious disease etiologies. Congenital CMV is a major, if not most frequent, cause of neonatal infectious disease-associated NDI in high-income countries. While there are data gaps in LMICs, accumulating evidence suggests CMV will also be a major contributor to congenital/neonatal NDI. It would seem prudent that some attempt at capturing other frequent causes of the endpoints studied, and either inclusion/exclusion criteria defined, or at least stratified analyses of the data by those other causes (if known).

All in all, this multi-country study has a reasonable likelihood of collecting a critical set of input data for determining if and how compelling the FPHVP is for developing vaccines or other interventions to protect against in utero, early and late onset iGBS.

Is the study design appropriate for the research question?

Partly

Is the rationale for, and objectives of, the study clearly described?

Yes

Are sufficient details of the methods provided to allow replication by others?

Yes

Are the datasets clearly presented in a useable and accessible format?

Yes

Reviewer Expertise:

vaccine and drug development and introduction

I confirm that I have read this submission and believe that I have an appropriate level of expertise to confirm that it is of an acceptable scientific standard, however I have significant reservations, as outlined above.

# Response

**Published:** 2021-05-26

**Authors:** Simon Procter

We thank the reviewer for their comments, our responses to the two specific points raised are below.

**(1) Sample size and power calculation: this description appears to describe an approach for determining whether there is a statistically significant difference between the iGBS survivors group and the matched non-iGBS control children for certain specific outcomes. That there is a statistically significant difference between the two groups is certainly of some interest, but would seem not to be the critical input data for a FPHVP assessment. Rather it would seem that the critical information would be: 1) an absolute point estimate and confidence intervals there of of burden of disease outcomes that are the major value drivers in the FPHVP; and 2) the excess burden of disease (see point 2 below) in iGBS survivors that are the major value drivers in the FPHVP. Perhaps the data analyses and statistical methodology that support these two estimates, with confidence intervals, is in the protocol, but if not, the authors should be encouraged to add and describe those items.**

 Thank you for this comment, which highlights an important distinction. We note that whilst this burden analysis is intended to input to the FPHVP, it is part of a broader exercise that requires and incorporates multiple other data sources. Hence, this paper details the approach for just these 5 low- and middle-income country data inputs including primary descriptive analysis of these datasets and is not focused on the broader FPHVP analyses. To make this clearer we have adjusted some of the text in the introduction and discussion (changed text highlighted in italics below) and have also added a reference on the FPHV of vaccination.

 “In this paper, we present the protocol for a multi-country epidemiological study *, **coordinated by the London School of Hygiene & Tropical Medicine***. The main aim of the study is to estimate the risk of NDI and socioemotional behaviors in children who survived neonatal or infant iGBS. The study will also measure long-term health related quality of life (HRQoL) and economic costs that arise as a consequence of iGBS, as well as the acute costs of sepsis and meningitis in young infants. ***Beyond the direct estimation of these outcomes in our study population**,* this study is ***also*** part of a collaboration with the World Health Organization (WHO) to inform development of a ***full public health value (FPHV)*** proposition for maternal vaccines against GBS.” **[Introduction paragraph 5]**

 “ ***As well as the direct analysis of the data described in this protocol**,* findings from this study will ***also*** be used to update previous morbidity estimates of the ***global*** burden of infant iGBS disease (12), adding relevant data on long-term outcomes. This will include information to estimate lifetime disability, including risk of NDI, and societal impacts following GBS-related sepsis and meningitis. These additional morbidity estimates, combined with the previously published data on mortality and morbidity of pregnant and postnatal women and stillbirths, along with other literature, will serve as data inputs for both mother and infant GBS disease to update estimates of the overall public health burden of iGBS. Disease burden estimates will be translated into DALYs incurred based on the latest available epidemiologic data, while responses to the quality of life instruments combined with mortality data will be used to estimate QALYs lost. These estimates will be an important input feeding into future cost-effectiveness analyses.

***These*** findings will contribute to the ***WHO-led development of a full public health value proposition*** for GBS maternal immunization to inform strategic planning of GBS vaccine research, development, and future implementation…” **[Discussion paragraphs 6 & 7]**

 As noted by the reviewer, for this protocol paper we included information on the power calculation to demonstrate that we should be able detect a statistically significant difference in the primary outcome of our study given the available number of iGBS cases and our chosen matching ratio of unexposed controls.  In addition to the statistical analyses described in the manuscript, to inform the FPHVP we will estimate the global burden of invasive GBS disease that might be averted through future GBS vaccines, which depends on both the incidence of iGBS, the fatality rate and the risk of long-term sequelae amongst survivors. To achieve this we will combine all the available evidence in a multi-step estimation process by: 

 (i) performing a Bayesian evidence synthesis of all evidence to estimate the incidence of early infancy iGBS disease (the methodological approach for this step is described in a recently accepted paper *Gonçalves BP et al. PLOS Comp. Biol. 2021. Article in press.*);

 (ii) meta-analysis of the fatality risk in children who survive iGBS disease;

 (iii) meta-analysis of the risk of mild NDI, and risk of moderate/severe NDI following GBS sepsis and meningitis.

 Our study will provide data on the risk of moderate/severe NDI, and of mild NDI, that will be used (alongside other existing data) in step (iii) in this process.  

**(2)** **Inclusion and exclusion criteria: Again, perhaps the information is provided in the protocol; however, it is unclear what the inclusion and exclusion criteria of selecting iGBS survivors, and equally important the case controls, with respect to other potential causes of the endpoints being measured. For example, NDI is multifactorial, including both genetic and infectious disease etiologies. Congenital CMV is a major, if not most frequent, cause of neonatal infectious disease-associated NDI in high-income countries. While there are data gaps in LMICs, accumulating evidence suggests CMV will also be a major contributor to congenital/neonatal NDI. It would seem prudent that some attempt at capturing other frequent causes of the endpoints studied, and either inclusion/exclusion criteria defined, or at least stratified analyses of the data by those other causes (if known).**

 We agree that this was unclear. For iGBS survivors we include any children that meet the case definitions in table 2.  In all of the countries, except for South Africa, children born <32 weeks gestation are excluded from the GBS exposed and unexposed groups.  South Africa does not have any exclusions based on gestational age. For unexposed cohorts in Argentina, children with known genetic disease were excluded and in South Africa exclusion criteria included no hospitalisation for the first 3 months and no markers of sepsis.  No other exclusion criteria for the unexposed group was used in the other sites. We agree that clarity on the inclusion/exclusion criteria is fundamental, we have clarified this by revising table 2 to include definitions for unexposed (non-GBS) children and additional exclusion criteria related to gestational age. We also added the following sentence to the manuscript adding the after the case definition:

 “ **In Argentina, India, Kenya, and Mozambique iGBS children and GBS unexposed children born earlier than 32 weeks of gestation are excluded.  In South Africa, gestational age is not an exclusion criterion.**”

 For all study participants we collect data on prior medical history but this does not include the history of other neonatal infections. However, NDI that may result from these other infections, including CMV, will form part of the background risk of NDI in our study settings. While we are not collecting data on these other aetiological causes of NDI, if numbers allow we will examine the influence of other potential confounders such as prematurity, SES and maternal education using multivariable regression.
